# Special Issue: Travel and Tropical Medicine

**DOI:** 10.3390/tropicalmed6020053

**Published:** 2021-04-19

**Authors:** Harunor Rashid, Al-Mamoon Badahdah, Ameneh Khatami

**Affiliations:** 1 National Centre for Immunisation Research and Surveillance (NCIRS), The Children’s Hospital at Westmead, Westmead, NSW 2145, Australia; 2 Marie Bashir Institute for Infectious Diseases and Biosecurity, School of Biological Sciences and Sydney Medical School, University of Sydney, Westmead, NSW 2145, Australia; 3 Discipline of Child and Adolescent Health, Faculty of Medicine and Health, The University of Sydney, Westmead, NSW 2145, Australia; ameneh.khatami@health.nsw.gov.au; 4 Department of Family and Community Medicine, Faculty of Medicine in Rabigh, King Abdulaziz University, Jeddah 22252, Saudi Arabia; ambadahdah@kau.edu.sa; 5 Department of Infectious Diseases and Microbiology, The Children’s Hospital at Westmead, Westmead, NSW 2145, Australia

Historically, travel is known to be associated with an amplified risk of acquisition and transmission of infectious diseases, including pandemics. In his travelogue, “Rihla”, Moroccan explorer Ibn Battutah record that his team contracted a febrile illness, most likely malaria, while in Kuzestan (Iran). Battutah keenly observed that “visitors to these countries in the hot season generally suffer from fever, as happens also in Damascus and other cities which have abundant waters and fruits”. He narrowly escaped the mediaeval black death of 1348 in Syria on his journey to Mecca for the Hajj pilgrimage [1]. However, compared to mainstream specialties of medicine, there is paucity of research in the field of travel medicine.

In this Special Issue, we present a suite of publications on various aspects of travel medicine ranging from refugee and immigrant health to mass gathering medicine. Highlights of this Special Issue include a case report of melioidosis in the United States in a Filipino immigrant [2] and another report of five cases of histoplasmosis among film crew members who acquired the illness in Guatemala and presented in Australia [3]; although rare, both these infections are important in travel medicine and practitioners should be aware of these exotic infections during pre-travel advice sessions. Nicknamed a “great mimicker”, for imitating other chronic infections, melioidosis should be considered in travellers returning from the Asia-Pacific. Histoplasmosis is a possibility in travellers with histories of exposure to bird or bat droppings, especially if immunocompromised.

Antimicrobial resistance (AMR) is a serious concern for travellers; two publications explored two different aspects of AMR: an original study showed fewer than 5% of health care workers (HCWs) who knew about a standard clinical guideline on antibiotic prescription (e.g., NICE-CG69, Centor Criteria) practised it correctly for Hajj pilgrims [4] and, in a systematic review, Fouz and colleagues show AMR genes can be detected in wastewaters from mass gatherings [5]. Both these publications highlight the importance of considering AMR among travellers and their external environments.

Two original articles focussed on the health of Rohingyas, among the most persecuted minorities in the world. One article assessed health literacy and health status of Rohingya refugees before their exodus to Bangladesh in 2017, showing that the majority (70%) of the 192 deaths that occurred in 1634 families in the year before their migration to Bangladesh occurred in men and 44% were claimed to be due to homicide [6]. A focussed survey involving 670 infants aged < 2 months showed that about 15% of children had watery or purulent discharge from their eyes [7]; although the study design did not include establishment of microbiological diagnosis, given the high likelihood of sexually transmitted infections among Rohingya refugees [8], some of these eye symptoms could be due to gonococcal ophthalmia neonatorum and, thus, deserve public health attention.

Two other review articles looked at rather unique topics in travel medicine: a systematic review on the understanding of immigrant populations’ knowledge and attitude on the human papillomavirus (HPV) vaccine showed immigrants often lacked sufficient knowledge of HPV infections and some had negative attitudes towards vaccination [9] and another study described pathophysiology of disproportionate thrombotic tendency of COVID-19 and its implications for travellers, along with guidance based on the severity of COVID-19 and coagulopathy [10]. 

One original study focussed on Hajj pilgrims’ understanding and practice of hand hygiene [11] and another of meningococcal vaccination [12]. A cross-sectional study conducted during the 2019 Hajj identified that most pilgrims knew hand hygiene could prevent respiratory and gastrointestinal infections, but many pilgrims did not know about precise hand washing methods. A follow up study conducted during the 2020 Hajj amidst the COVID-19 pandemic showed no improvement and pilgrims’ mean hand hygiene knowledge score remained essentially unchanged (mean score 6.7 (±1.9) vs 6.4 (±1.35) of total 12) [11,13]. Another cross-sectional study conducted in 2017 and 2018 showed that 13.4% of Hajj pilgrims certainly missed meningococcal vaccination and another 4.8% were unsure about their vaccination status, which is a great concern [12]. 

Since 2000, when the quadrivalent meningococcal vaccination was made a Hajj visa-requirement, the uptake among overseas pilgrims ranged from 93% to 100% [12,14,15], but the vaccination rate among domestic pilgrims was lower (≤82%) [11,12,16,17]. A recent study with detailed breakdown on vaccination history has shown that, even among overseas pilgrims, the actual vaccine uptake is only 77%. At least 11% of vaccination certificates are fake and another 12.6% are dubious; in 0.5%, an incorrect vaccine (e.g., bivalent vaccine) is recorded, making the certificate non-valid [18]. Suboptimal vaccination rates have also been reported among HCWs at Hajj [19,20]. These findings forecast a serious problem that could arise following the implementation of “vaccination passports” to allow passengers to avoid official border restrictions and quarantine as part of the global COVID-19 control strategy [21]. Non-vaccination and use of false vaccination certificates to cross international borders would jeopardise global efforts to curb the pandemic. Stringent measures supervised by international public health observers are needed to ensure the safety of world health.
